# Human Metapneumovirus Pneumonia Precipitating Acute Respiratory Distress Syndrome in an Adult Patient

**DOI:** 10.7759/cureus.16434

**Published:** 2021-07-17

**Authors:** Dena H Tran, Muhammad Sameed, Ellen T Marciniak, Avelino C Verceles

**Affiliations:** 1 Internal Medicine, University of Maryland Medical Center Midtown Campus, Baltimore, USA; 2 Pulmonary and Critical Care Medicine, University of Maryland School of Medicine, Baltimore, USA

**Keywords:** human metapneumovirus (hmpv), ards (acute respiratory distress syndrome), severe respiratory failure, immunocompromised patient, pulmonary and critical care medicine

## Abstract

Acute respiratory distress syndrome (ARDS) is often due to direct lung injury, trauma, surgery, or infection. Making a definitive diagnosis may be difficult initially, as clinical manifestations are nonspecific until the disease progresses. We present a case of human metapneumovirus (hMPV) pulmonary infection precipitating ARDS.

A 51-year-old woman presented with one week of pleuritic chest pain, dyspnea, wheezing, subjective fever, and productive cough prior to presentation. Her medical history was significant for human immunodeficiency virus (HIV) with an unknown CD4 count and viral load, pulmonary sarcoidosis, asthma, and being an active smoker. On admission, the patient was dyspneic and using accessory muscles to breathe. She was afebrile and hypotensive. Physical examination revealed bilateral diffuse crackles. Her white blood cell (WBC) count was 7.7 K/mcL. A chest radiograph demonstrated bilateral lung opacifications suggestive of pneumonia, possibly *Pneumocystis* jiroveci pneumonia (PJP). Broad-spectrum antibiotics, including PJP treatment, corticosteroids, and fluids, were started. The patient received approximately 4 liters of intravenous fluids; yet, she remained hypotensive and required norepinephrine. Chest computed tomography (CT) demonstrated bilateral consolidations. Arterial blood gas (ABG) showed a partial pressure of oxygen (PaO_2_) of 55 mmHg. The patient was intubated for acute hypoxemic respiratory failure and had a PaO_2_/fraction of inspired oxygen (FiO_2_) < 100. Repeat ABG within 12 hours showed a potential of hydrogen* (*pH) of 7.34, partial pressure of carbon dioxide (pCO_2_) of 42 mmHg, and a PaO_2_ of 130 mmHg. Bronchoalveolar lavage revealed only hMPV. The patient was managed supportively and extubated three days later. She was discharged home without oxygen requirement.

hMPV causes respiratory infections, most commonly in the extremes of age and immunocompromised patients. The treatment is supportive. Our patient developed acute hypoxemic respiratory failure secondary to an hMPV infection. hMPV pneumonia should be considered as a differential diagnosis in patients with severe respiratory illness and ARDS in order to promote antibiotic stewardship.

## Introduction

Human metapneumovirus (hMPV) was first discovered in 2001 and is more common among the pediatric population with respiratory disease [1]; however, elderly and immunocompromised patients are also vulnerable to this virus [2-3]. hMPV is a single-stranded, enveloped ribonucleic acid (RNA) virus that belongs to the *Paramyxovirus* family and is closely related to parainfluenza viruses, including influenza A and respiratory syncytial virus (RSV). hMPV infection in young children presents with mild flu-like symptoms and is a self-limiting illness [4]. However, hMPV infection in the older population and the immunocompromised may lead to a more severe illness course that can progress to acute respiratory distress syndrome (ARDS) [5-8]. We present a case of hMPV precipitating ARDS in an adult population.

## Case presentation

A 51-year-old woman presented with a one-week history of pleuritic chest pain, shortness of breath, wheezing, and subjective fever associated with a productive cough with yellow sputum in June. She had been in a rehabilitation facility where her roommate was exhibiting signs of upper respiratory illness. Her medical history was significant for human immunodeficiency virus (HIV), obstructive sleep apnea (OSA), and intravenous drug use. Her home medications included albuterol, rilpivirine, dolutegravir, and methadone. She had a current 15-pack year smoking history and was on a methadone program. She had a documented allergic reaction to sulfa antibiotics.

**Figure 1:**
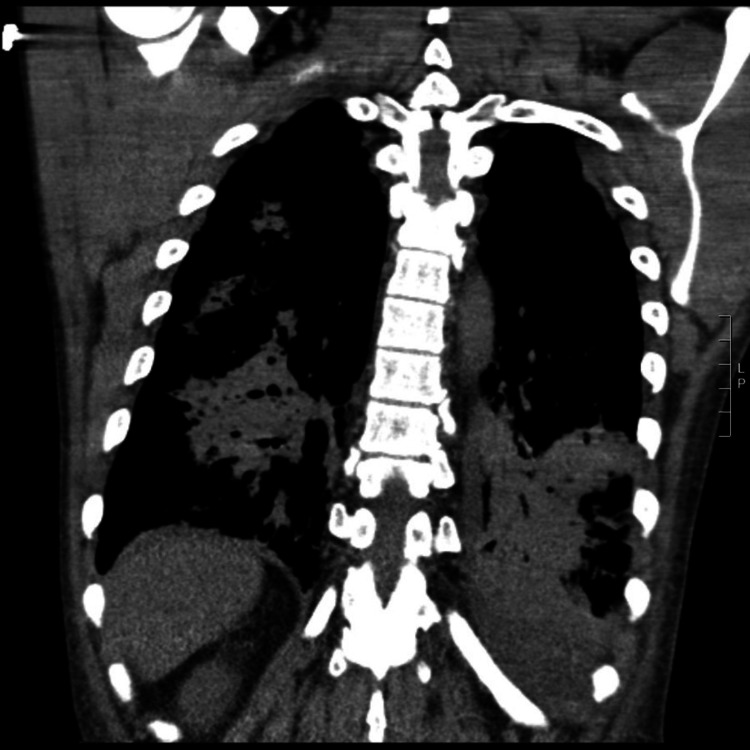
Computed Tomography (CT) of the Chest CT of the chest shows bilateral consolidations, concerning for bilateral multifocal pneumonia secondary to human metapneumovirus.

**Figure 2:**
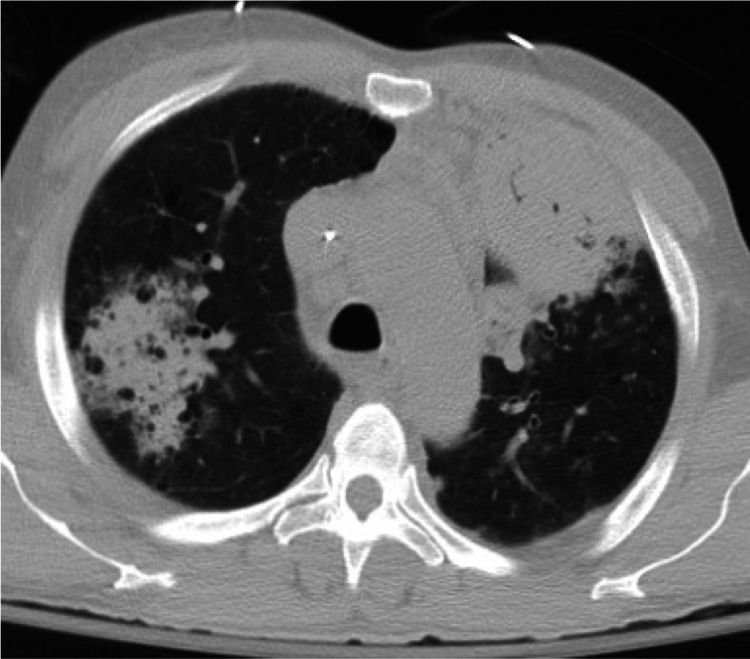
CT of the Chest CT of the chest (lung window) illustrating multifocal pneumonia secondary to human metapneumovirus (hMPV).

In the emergency department, the patient was afebrile (99.0°F) with a heart rate of 76 beats/min, blood pressure of 67/33 mmHg, respiratory rate (RR) 14 breaths/min, and a saturation percentage of oxygen in the blood (SpO_2_) of 94% on 5 liters/min oxygen supplementation via a nasal cannula. Her complete blood count was significant for a leukocyte count of 7.7 K/mcL with 21% bands; other cell counts were unremarkable. Her serum chemistry panel showed a low bicarbonate of 19 mmol/L and an elevated lactate of 4.5 mmol/L. She was treated for presumptive sepsis with 30 mL/kg of balanced crystalloids (3 liters), broad-spectrum antibiotics (piperacillin/tazobactam and vancomycin), and supplemental oxygen via nasal cannula. A chest x-ray revealed bilateral lung opacifications suggestive of pneumonia. Computed tomography (CT) scan of the chest confirmed bilateral consolidations, concerning for bilateral multifocal pneumonia (Figures 1-2). The patient continued to be hypotensive despite an additional one liter of normal saline and was started on norepinephrine infusion. The patient became hypoxic with a SpO_2_ of 80% despite being on 6 liters/min nasal cannula oxygen, requiring intubation. She had an arterial blood gas (ABG) that showed a potential of hydrogen (pH) of 7.37, partial pressure of carbon dioxide (pCO_2_) of 41 mmHg, and a partial pressure of oxygen (PaO_2_) of 55 mmHg (PaO_2_/fraction of inspired oxygen (FiO_2_)​​​​​​​ < 100). Intravenous methylprednisolone and clindamycin were added to cover for *Pneumocystis *jiroveci pneumonia (PJP). The patient was admitted to the intensive care unit (ICU) for further management.

In the ICU, the patient was on volume assist control ventilation with a tidal volume (Vt) of 400 ml, respiratory rate (RR) 24/min, and a positive end-expiratory pressure (PEEP) 7 cm of H_2_O, FiO_2_ 40%. The patient was ventilated following the ARDS protocol (targeting Vt 6 - 8 mL/kg of predicted body weight, plateau pressure ≤ 30 cm of H_2_O). Further workup revealed a normal lactate dehydrogenase (LDH). Blood cultures collected in the emergency department were negative, troponin was elevated at 1.2 ng/mL, lactate trended down to 1.7 mmol/L, CD4 count was 85, and HIV viral load was undetectable. Within the next 12 hours, the patient was weaned off of norepinephrine. A repeat ABG showed a pH of 7.34, PaCO_2_ of 42 mmHg, and PaO_2_ of 103 mmHg. A bronchoscopy and bronchoalveolar lavage (BAL) was performed and tested for respiratory viruses, PJP antigen, gram stain, bacterial, and fungal cultures. The BAL sample was positive for the only hPMV. The patient was successfully extubated three days after admission. Her echocardiogram showed a normal left ventricular ejection fraction of 60%. The patient was ultimately discharged home without oxygen requirement. 

## Discussion

Acute respiratory distress syndrome (ARDS) was first described in 1967 as a syndrome with hallmarks of refractory hypoxia, diffuse radiographic opacities, and diffuse alveolar damage on histology [9]. ARDS cannot be diagnosed by a single laboratory test; neither is it associated with a specific etiology. The American-European Consensus Conference (AECC) formulated the first working definition of ARDS that allowed for epidemiological and translational research [10]. The Berlin definition later refined the diagnostic criteria for ARDS by defining acute onset as seven days and is defined as mild, moderate, and severe ARDS with varying ranges of the PaO_2_/FiO_2_ ratio. Because no biomarker exists to diagnose ARDS, it is oftentimes difficult to diagnose ARDS until the disease progresses [11]. Villar et al. demonstrated that a PaO_2_ response after a 24-hour period of standardized ventilator settings (PEEP ≥ 10 and FiO_2_ ≥ 0.50) allows for dynamic segregation of ARDS patients into several categories [12]. 

Our patient developed acute hypoxemic respiratory failure secondary to hMPV infection. As all other infectious workups were negative without other obvious causes of pulmonary injury, hMPV infection was the presumptive cause of respiratory failure. There have been limited reports showing hMPV causing ARDS [13-15]. In our case, the patient met the Berlin criteria and had a PaO_2_/FiO_2_ ratio < 300, supporting the diagnosis of ARDS. Huppert et al. demonstrated increased pulmonary edema fluid accumulation in ARDS due to lung inflammation as a result of increased capillary permeability [16]. In our case, our patient received approximately 4 liters of intravenous crystalloids, likely precipitating ARDS. 

hMPV causes respiratory infections, commonly in the extremes of age and in the immunocompromised. The treatment is supportive. ARDS may initially be misdiagnosed as, or thought to be caused by, bacterial pneumonia, and patients are started on antibiotics. Viruses, particularly influenza A, can also cause ARDS, especially if there is an underlying immunodeficiency [17]. Vidaur et al. performed a 10-year retrospective analysis on the association between hMPV and severe community-acquired pneumonia and found that 92.8% of patients were infected with hMPV within the first half of the year [14]. It is important to test for a respiratory viral panel outside of the influenza season, and hMPV should be considered in patients with ARDS to allow for antibiotic de-escalation to prevent antibiotic resistance.

## Conclusions

Human metapneumovirus (hMPV) causes respiratory infections, commonly in the extremes of age and in immunocompromised patients. The treatment is supportive. Our patient developed acute hypoxemic respiratory failure secondary to an hMPV infection in June. A respiratory viral panel should be tested outside of the influenza season. hMPV pneumonia should be considered in patients within the first half of the year who present with severe respiratory illness in order to promote antibiotic stewardship.
